# Morphological and molecular characterization of *Tatera indica* Hardwicke 1807 (Rodentia: Muridae) from Pothwar, Pakistan

**DOI:** 10.1515/biol-2022-0063

**Published:** 2022-06-15

**Authors:** Amber Khalid, Amjad Rashid Kayani, Muhammad Sajid Nadeem, Muhammad Mushtaq, Mirza Azhar Beg

**Affiliations:** Department of Zoology, Pir Mehr Ali Shah Arid Agriculture University, Rawalpindi, Pakistan

**Keywords:** *Tatera indica*, genetics, cytochrome *b*, molecular DNA, morphometry

## Abstract

Genus Tatera is comprised of 12 species, but only one species, *Tatera indica*, exists in Asia. *T. indica* is considered an agricultural pest and host of a few zoonotic diseases. However, the data on morphological characteristics are published, but the aspect of molecular characterization is lacking to confirm the status of subspecies in most of the Asian countries including Pakistan. Therefore, the present study is the first study on morphological and molecular characterization of *T. indica* to get a phylogenetic relationship with the population of *T. indica* in Pakistan. Almost all the morphometric, cranial, and dental values of *T. indica* were lower in the present study as compared to the specimen from Iran, Syria, and Turkey. The molecular analysis indicated the presence of sub-species of *T. indica*. Interestingly, the cytochrome *b* gene sequence showed more resemblance to the Iranian rat than the previously reported sequence of a Pakistani *T. indica.* Iran shares a border with Pakistan, and migration between the two countries could be a possible reason. The resembling morphometric data on Iranian rats also explain this phenomenon. The present study found, although minor, evidence of the presence of sub-species even within Pakistan. Unfortunately, the previously submitted sequence from Pakistan was not supplemented with morphometric data and the exact capturing site of the rat. Therefore, further molecular and morphometric data from other regions are required to confirm the presence of sub-species.

## 1 Introduction

The members of the genus *Tatera* are common in the cultivated areas. Out of 12 species, 11 are found in Africa and only one (*Tatera indica*) in Asia [1,2]. *T. indica* is distributed in Pakistan, Afghanistan, Iraq, Nepal, India, Kuwait, Sri Lanka, Syria, and Turkey [3,4,5,6,7,8,9]. The distribution of *T. indica* in Pakistan is known poorly. In Pakistan, its presence has been reported throughout Punjab except for the foothills of Murree, parts of Khyber Pakhtunkhwa province, and Sindh and Baluchistan provinces [6,10].

Chevret and Dobigny declared *Tatera* a polyphyletic taxon based on the genomic DNA [11]. They proposed that the migration occurs from Asia to Africa, unlike other gerbiline rodents. Ancestors of *T. indica* belonged to Africa, and they voyaged to Asia by some land bridges. The subspecies status of *T. indica* is still controversial. In Iran, four different subspecies, namely *T. indica persica*, *T. indica scansa*, *T. indica monticola*, and *T. indica bailwardi*, were reported earlier [12]. However, later on, only two subspecies *T. indica* and *T. indica teaniura* were recognized in Iran [13]. Furthermore, Mirshamsi et al. found that there is no discrimination between the southern and northern populations of *T. indica* in Iran [14]. Mohammadi and Pervaizi found that two phenotypically different *T. indica*, i.e., buff-black and buff-brown, are not subspecies molecularly [9]. However, in Pakistan, only buff-browns were reported to exist. No record of any subspecies of *T. indica* was found in Pakistan.

Although morphometry is a useful, cheaper, and robust tool for identifying and classifying animals, molecular analysis is needed to confirm species identification and determine intraspecific variation. The proper identification and estimation of the diversity of *T. indica* are pre-requisite for the control of this agricultural pest and a host of leishmaniasis. Therefore, the present study was designed to perform morphometric and molecular characterization of *T. indica* from Pothwar, a subtropical area in the province of Punjab, Pakistan.

## 2 Materials and methods

### 2.1 Study site and sample collection

A total of 120 samples of *T. indica* were collected from different active colonies of rats in Pothwar, a semi-arid subtropical plateau in the north of Punjab province of Pakistan. It covers a 23,160 sq km area and stretches from latitude 32°10 to 34°9N and longitude 71°10–73°55E with an altitude of 305–610 m above sea levels. The specimens were collected using snap traps from dusk to dawn by baiting them with bread dipped in vegetable oil. Collected rats were brought to the Laboratory of Ecology and Wildlife PirMehr Ali Shah, Arid Agriculture University, Rawalpindi.


**Ethical approval:** The research related to animal use has complied with all the relevant national regulations and institutional policies for the care and use of animals and was approved by the Ethical Committee of PirMehr Ali Shah, Arid Agriculture University, Rawalpindi.

### 2.2 Morphological study

#### 2.2.1 Body measurements

A total of 85 specimens were subjected to morphometric analysis. Each specimen was weighed using a digital balance (Model: TX323L, MedigeneSdn. Bhd., Malaysia). Standard methods of measurement were used to measure six external morphological features, namely total body length (TBL), head and body length (HBL), tail length (TL) from the rump, ear length (EL) from basal notch to the distal tip of the head and left hindfoot length (HFL) without claw using a ruler, compass, and/or an analog vernier caliper and recorded in a tabulated form [6].

#### 2.2.2 Skull measurements

The skull was prepared by detaching the ligaments of the head from the neck using scissors and forceps. The detached head was cleaned by removing the flesh and eyes from the eye socket. It was then placed in the 2% sodium hydroxide (NaOH) solution. When all the remaining flesh was separated, the cleaned skull was preserved in airtight bags or the small boxes containing cotton pads. Each skull was tagged with the original field number and other specific information for identification. Eight measurements of skull bones, including nasal length (NL), zygomatic breadth (ZB), greatest skull length (GSL), mandible length (ML), upper molar tooth row length (UMRL), lower molar tooth row length (LMRL), and least interorbital breadth, were measured using the standard methods mentioned above.

### 2.3 Genomic and phylogenetic analysis

The phenol-chloroform (Organic) method was used to extract DNA from ∼0.25 g of tail tissue of each specimen (*n* = 13). Extracted DNA was evaluated for quality and quantity by using standard methods, and pellets were stored at 4°C. DNA was amplified for the mitochondrial cytochrome *b* (Cyt-*b*) gene with the forward primer UNFOR403 (5′-TGAGGACAAATATCATTCTGAGG-3′) and the reverse primer UNREV1025 (5′-GGTTGTCCTCCAATTCATGTTA-3′) through specific conditions optimized for the polymerase chain reaction (PCR) (Model: 865061CL, Galaxy XP ClearLine Thermal Cycler, China). The primers were selected as described in Kent and Norris [15]. Amplification of PCR product was confirmed on ethidium bromide-stained 2% agarose gel visualized under UV.

The purified PCR products of the Cyt-*b* gene were sequenced directly, and the sequences were aligned by using Mesquite and MEGA X 10.1 Software. The sequences were blasted in GenBank, and a phylogenetic tree was constructed using MOLE-BLAST to find out the closest database neighbors (https://blast.ncbi.nlm.nih.gov/moleblast/moleblast.cgi). The aligned sequences were also compared with already published sequenced mitochondrial Cyt-*b* of *T. indica* (GenBank Accession No.: KP001566 and AJ430563). Tajima’s Neutrality test was conducted using MEGA X 10.1 software. Estimation of nucleotide composition and the maximum likelihood were also calculated using the above-mentioned software.

## 3 Results

### 3.1 Physical appearance

The dorsal side body color is reddish-brown to fawnish, while the ventral side of the body is creamy white in color. Feet are naked and are hairless. The forefoot has four toes with a thumb. The hindfoot has five digits. Female individuals have four pairs of mammae. The animal’s tail is approximately half of the length of the body. The tail has a tuft of black hairs at the end, and a brown band is present on the sides of the tail. Ears are also naked and elongated.

### 3.2 Dietary habits

*T. indica is* an omnivorous species. It predominately consumed plant matter in its diet along with animal matter. When the supply of insects increases during the rainy season, they feed on insects.

### 3.3 Habitat and predation

*T. indica* is successfully adopted in different habitats. They can occupy habitats if there is an abundant supply of food and frequent through irrigation canals. However, they avoid cold temperatures. They primarily preyed on by birds of prey, especially owls.

### 3.4 Morphological diversity

#### 3.4.1 Body measurements

The body color of all the samples was brown. The body weight (BW) of rats ranged from 55.4 to 154.9 g (118.3 ± 25.4 g). The TBL of the adult animal was 240–350 mm (350.1 ± 24.5 mm). HBL was 110–180 mm (142.3 ± 19.1 mm). TL was 120–200 mm (164.2 ± 16. mm5). The EL was 10–25 mm (20.2 ± 2.8 mm). HFL was 30–40 mm (35.8 ± 2.1 mm) (Table 1). TBL, HFL, HBL, TL, and EL were used as distinguishing features of *T. indica* from other rat species in the area (Table 1)

**Table 1:** Morphometric measurements of *T. indica* (*n* = 85)

Character	Mean ± SD	Min	Max
BW (g)	118.3 ± 25.4	55.4	154.9
TBL (mm)	350.1 ± 24.5	240	350
HBL (mm)	142.3 ± 19.1	110	180
TL (mm)	164.2 ± 16.5	120	200
HFL (mm)	35.8 ± 2.1	30	40
EL (mm)	20.2 ± 2.8	10	25

BW: body weight; TBL: total body length; TL: tail length; HBL: head and body length; HFL: hindfoot length; EL: ear length.

#### 3.4.2 Cranial measurements

The GSL of rats ranged from 36.5 to 97.2 mm (39.6 ± 3.1 mm). The diameter of the auditory bulla was 3.4–5.9 mm (5.1 ± 1.1 mm). Depth of braincase and breadth of braincase were 12.2–14.2 mm (13.4 ± 1.0 mm) and 14.6–18.8 mm (17.3 ± 2.3 mm), respectively. ZB was 22.0–22.1 mm (27.9 ± 0.01 mm). The EL was 10.0–25.0 mm (20.2 ± 2.8 mm), while NL was 14.9–17.5 mm (16.3 ± 1.0 mm). LMR and UMRL were 5.2–6.0 mm (5.8 ± 0.3 mm) and 5.8–6.4 mm (6.1 ± 0.2 mm), respectively. The ML was 19.33–20.81 mm (19.9 ± 0.6 mm), while the first molar length was 3.19–3.7 mm (3.4 ± 0.2 mm) (Table 2).

**Table 2:** Cranial measurements of *T. indica*

Measurements (mm)	*N*	Mean ± SD	Min	Max
GSL	65	39.6 ± 0.3	36.4	97.2
BBC	64	17.3 ± 2.3	14.6	18.8
DBC	68	13.4 ± 1.0	12.2	14.2
ZB	61	27.9 ± 0.01	22.0	22.1
NL	66	16.3 ± 0.9	14.9	17.5
ABD	66	5.1 ± 1.1	3.4	5.9
IFL	65	7.5 ± 0.1	7.4	7.5
UMRL	66	6.1 ± 0.2	5.8	6.4
MIL	66	3.4 ± 0.2	3.2	3.7
IOB	66	6.7 ± 0.2	5.5	6.7
ML	66	19.9 ± 0.6	19.3	20.8
LMRL	66	5.8 ± 0.3	5.2	6.0

GSL: greatest skull length, BBC: breadth of braincase, DBC, depth of braincase, ZB, zygomatic breadth, NL, nasal length, ABD, auditory bulla diameter, FL, incisive foramen length, UMRL, upper molar tooth row length, MIL, first molar length, IOB, interorbital breadth, ML, mandible length LMRL lower molar tooth row length.

### 3.5 Genetic diversity

To determine the genetic diversity between the two populations, nucleotide sequences were compared using BLAST, Clustal W, and MEGA software. The sequences from all 13 samples were almost similar. All the sequences were submitted to the GenBank (Accession numbers: BankIt2492881 AK9789-13 MZ852449 to BankIt2492881 AK9789-1 MZ852461). Supplementary files 1 and 2 are attached as sequence alignment data and individual sequences of samples.

#### 3.5.1 Nucleotide sequence BLAST analysis

The obtained sequence of the Cyt-*b* gene from the selected rat species was subjected to BLAST (https://blast.ncbi.nlm.nih.gov/Blast.cgi). It brought two matched sequences of *T. indica* from Iran (KP001566) and Pakistan (AJ430563). Our sequences had more similarities with the Iranian (97.11%) sample than Pakistani (96.43%).

#### 3.5.2 Phylogenetic tree based on nucleotide sequence

**Figure 1:**
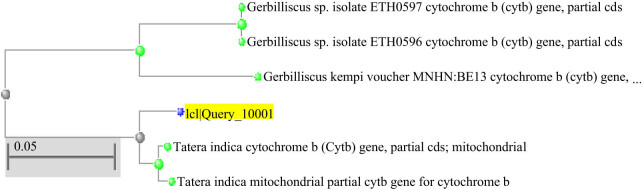
Phylogenetic tree constructed by MOLE-BLAST where ICII Query_10001 was resulted in the sequence of Cyt-*b* gene.

The phylogenetic tree was also constructed using MOLE-BLAST (https://blast.ncbi.nlm.nih.gov/moleblast/moleblast.cgi). The phylogram has shown 0.05 genetic diversity indicating a close relationship with the Cyt-*b* of already published sequences of the Cyt-*b* gene of *T. indica.* The present molecular analysis showed a relationship with Gerbilliscus (Figure 1).

#### 3.5.3 Maximum likelihood estimate of substitution matrix

The substitution rates and patterns were estimated using Tamura-Nei (1993) model [16]. The rates of different substitutions are shown in Table 3. The analysis involved 15 nucleotide sequences of the Cyt-*b* gene, 13 sequences from the present study, and 2 already published sequences. The estimated nucleotide frequencies were *A* = 30.42%, T/U = 30.77%, *C* = 27.49%, and *G* = 11.31%. The maximum Log-likelihood for this computation was −979.044.

**Table 3:** Maximum likelihood estimate of substitution matrix

	A	T/U	*C*	*G*
A	—	*2.74*	*2.45*	**7.34**
T/U	*2.71*	—	**26.01**	*1.01*
C	*2.71*	**29.11**	—	*1.01*
G	**19.73**	*2.74*	*2.45*	—

A: adenine, T: thymine, U: uracil, G: guanine, C: cytosine.

The bold values represent the rates of different transitional substitutions, and the italics represent the transversions substitutions.

#### 3.5.4 Tajima’s neutrality test

Tajima’s neutrality test was conducted using MEGA X10.1 software for 15 nucleotide sequences of the Cyt-*b* gene of *T. indica.* The nucleotide diversity was investigated for the nucleotide sequences of the Cyt-*b* gene had given 26 segregating sites, including nucleotide diversity of 0.009370 (Table 4).

**Table 4:** Tajima’s neutrality test based on Cyt-*b* gene of *Tatera indica* using MEGA X 10.1

No. sequences “*m*”	No. segregating sites “*S*”	ps = *S*/*n*	Θ = ps/*a*1	Nucleotide diversity (π)	Tajima’s test statistic “D”
15	26	0.041868	0.012876	0.009370	−1.136164

*m*: number of sequences, *n*: total number of sites, *S*: number of segregating sites.

## 4 Discussion

Almost all research studies on morphometry of *T. indica* were of Iranian origin [9,14,17]. However, only two studies reported molecular characterization of *T. indica* in Iran and Pakistan [9,11]. In Pakistan, most of the studies on *T. indica* reported its status, habits, and susceptibility as a disease vector. Except for the BW of *T. indica*, the published morphometric data were not available from Pakistan [18].

Most of the studies worldwide distinguished the coloration of *T. indica* as brown and black but did not report morphometrics separately. All the specimens captured in the present study were brown. Almost all the morphometric values of *T. indica* were lower in the present study as compared to the previous studies [8,9,19]. The most closed measurements were reported from Iranian *T. indica* that was captured from southeastern Iran and adjoining areas of Pakistan [16,20]. Although the mean values of BW corresponded to the previous studies, the minimum BW of rats in the present study was lower (55.42 g) than in all previous reports [8,9,19]. However, *all studies found that T. indica from Syria was the heaviest (196*–*356 g)* [19]. The minimum and maximum TBL (240–350 mm) in the present study were the lowest compared to the previous studies. The values of TBL of the Turkish rats were the highest (368–480 mm) among all studies [8]. The values of HBL were also lower in the present study (110–180 mm) as compared to those reported in Iranian (160–300 mm) and Syrian (171–215 mm) studies [9,19]. TL, HFL, and ER were also the lowest in the present study compared to the previous reports from Iran, Syria, and Turkey [8,9,19]. Similarly, almost all the cranial and dental measurements were measured least in the present study compared to the previous reports [8,9,19]. The cranial and dental measurements being positively correlated with body size have been used traditionally for the estimation of body size in rodents [21]. The smaller body size in the present study well explained the lowest cranial and dental measurements.

Body size in small mammals is determined by season, climate, latitude, and food availability and may fluctuate with these factors [17,18,20]. The *T. indica* captured from northeastern Iran at a higher altitude and cold climate had increased body size than from southeastern regions [20,22,23]. The temperature has a negative association with the body size of these mammals to retain heat in cold climates and emit heat in hot climates. Syria and Turkey are at a higher latitude than the Pothwar region and experience lower temperatures than the Pothwar. The Pothwar region is a sub-tropical plateau exhibiting seasonal temperature fluctuation, but the temperature rarely goes near or below freezing point, especially in areas at lower altitudes. Furthermore, the temperature in summer reaches 45°C or above. Along with these climatic factors, food availability in the area is an important covariate to consider for the variable body size of rodents [20].

The molecular analysis of the Cyt-*b* gene showed single-nucleotide polymorphisms compared with already published literature submitted in GENBANK. BLAST analysis showed that the sequence of the Cyt-*b* gene showed more resemblance to the Iranian sample (Accession No. KP001566) than the previously reported sequence of a Pakistani *T. indica* (Genbank Accession No. AJ430563). Iran shares a border with Pakistan, and migration between the two countries could be a possible reason. The resembling morphometric data on Iranian rats also explain this phenomenon. The present study found, less evidence of presence of sub-species within Pakistan. However, morphometric data along with molecular characterization is novel information reported here. The subspecies status of *T. indica* is still controversial. In Iran, four different subspecies, namely *T. indica persica*, *T. indica scansa*, *T. indica monticola*, and *T. indica bailwardi*, were reported earlier (Ellerman 1948). However, later on, only two subspecies namely *T. indica indica* and *T. indica teaniura* were recognized in Iran [13]. Furthermore, Mirshamsi et al. found that there is no discrimination between the southern and northern populations of *T. indica* in Iran [14]. Mohammadi and Pervaizi found two phenotypically different *T. indica*, i.e., buff-black and buff-brown, but molecularly these are not subspecies [9]. It is also not possible to explain great morphometric variations in the *T. indica* from other Asian regions (Syria and Turkey) due to the unavailability of genetic data in those studies. The morphometric variations alone could be explained only due to climatic pressure. Molecular approaches are now often used to complement morphological taxonomic methods for the identification of the species, as species identification is the first step in any kind of biological research. Thus, molecular data including numerous new genes yield true biological diversity. The results thus proved that conventional methods of identification by morphometry should be accomplished with molecular techniques to find out the original intraspecific diversity that could be helpful for the species-specific pest management.

## 5 Conclusion

Almost all the morphometric, cranial, and dental values of *T. indica* were lower in the present study compared to the previous studies. The molecular analysis indicates the presence of sub-species of *T. indica*. However, further molecular and morphometric data from other regions are required to validate the presence of sub-species.

## Supplementary Material

Supplementary Material 1

Supplementary Material 2
