# Supplementary material for: Morphological and molecular characterization of Tatera indica Hardwicke 1807 (Rodentia: Muridae) from Pothwar, Pakistan
Source: Open Life Sci. 2022 Jun 15;17(1):610–5. doi: 10.1515/biol-2022-0063 (PMC9202534; doi:10.1515/biol-2022-0063)
Supplement: Supplementary Material 1 [file biol-2022-0063-Supplementary-1.docx]

>AK9789-13

GG------------CAGAAGGA----------TACATGCTTACTCTCAGCCATTCCATACATTGGAACAACAATCGTAGAATGAATCTGAGGCGGCTTTTCTGTAGATAATGCCACACTAACACGCTTTTTCGCATTCCATTTTATTCTACCATTTATCATTACTGCTATAGTCCTAGTTCACCTGCTATTTCTCCATGAAACAGGATCTAACAACCCTATAGGCATCAACTCTGACGCAGACAAAATTCCATTCCACCCATATTACACAATCAAAGACCTCTTAGGAGTAATTTTATTAATTTCATTATTCATACTTATAGTCCTATTCTCTCCTGACCTTCTAGGTGACCCCGACAATTACACACCAGCTAACCCACTCAATACACCCCCTCATATCAAGCCAGAATGATACTTCCTATTTGCTTACGCCATTCTACGATCCATCCCTAACAAATTAGGAGGAGTTTTAGCCCTCATTCTTTCCATCCTCATCCTAATAATTTTACCTCTTACCCACACATCAAAACAACGAAGCTTAATATTCCGACCAATCTCCCAATTTATTTTCTGACTTTTAGTAGCCAACTTACTAATCCTAACATGAATTGGAGGACAACCA

>AK9789-12

GG------------CAGAAGGA----------TACATGCTTACTCTCAGCCATTCCATACATTGGAACAACAATCGTAGAATGAATCTGAGGCGGCTTTTCTGTAGATAATGCCACACTAACACGCTTTTTCGCATTCCATTTTATTTTACCATTTATCATCACTGCTATAGTCCTAGTTCACCTGCTATTTCTCCATGAAACAGGATCTAACAACCCTATAGGCATCAACTCTGACGCAGACAAAATTCCATTCCACCCATATTACACAATCAAAGACCTCTTAGGAGTAATTTTATTAATTTCATTATTCATACTTATAGTCTTATTCTCTCCTGACCTTCTAGGTGACCCCGACAATTACACACCAGCTAACCCACTCAATACACCCCCTCATATCAAGCCAGAATGATACTTCCTATTTGCTTACGCCATTCTACGATCCATCCCTAACAAATTAGGAGGAGTTTTAGCCCTCATTCTTTCCATCCTCATCCTAATAATTTTACCTCTTACCCACACATCAAAACAACGAAGCTTAATATTCCGACCAATCTCCCAATTTATTTTCTGACTTTTAGTAGCCAACTTACTAATCCTAACATGAATTGGAGGACAACCA

>AK9789-11

GG------------CAGAAGGA----------TACATGCTTACTCTCAGCCATTCCATACATTGGAACAACAATCGTAGAATGAATCTGAGGCGGCTTTTCTGTAGATAATGCCACACTAACACGCTTTTTCGCATTCCATTTTATTCTACCATTTATCATTACTGCTATAGTCCTAGTTCACCTGCTATTTCTCCATGAAACAGGATCTAACAACCCTATAGGCATCAACTCTGACGCAGACAAAATTCCATTCCACCCATATTACACAATCAAAGACCTCTTAGGAGTAATTTTATTAATTTCATTATTCATACTTATAGTCTTATTCTCTCCTGACCTTCTAGGTGACCCCGACAATTACACACCAGCTAACCCACTCAATACACCCCCTCATATCAAGCCAGAATGATACTTCCTATTTGCTTACGCCATTCTACGATCCATCCCTAACAAATTAGGAGGAGTTTTAGCCCTCATTCTTTCCATCCTCATCCTAATAATTTTACCTCTTACCCACACATCAAAACAACGAAGCTTAATATTCCGACCAATCTCCCAATTTATTTTCTGACTTTTAGTAGCCAACTTACTAATCCTAACATGAATTGGAGGACAACCA

>AK9789-10

GG------------CAGAAGGA----------TACATGCTTACTCTCAGCCATTCCATACATTGGAACAACAATCGTAGAATGAATCTGAGGCGGCTTTTCTGTAGATAATGCCACACTAACACGCTTTTTCGCATTCCATTTTATTCTACCATTTATCATTACTGCTATAGTCCTAGTTCACCTGCTATTTCTCCATGAAACAGGATCTAACAACCCTATAGGCATCAACTCTGACGCAGACAAAATTCCATTCCACCCATATTACACAATCAAAGACCTCTTAGGAGTAATTTTATTAATTTCATTATTCATACTTATAGTCTTATTCTCTCCTGACCTTCTAGGTGACCCCGACAATTACACACCAGCTAACCCACTCAATACACCCCCTCATATCAAGCCAGAATGATACTTCCTATTTGCTTACGCCATTCTACGATCCATCCCTAACAAATTAGGAGGAGTTTTAGCCCTCATTCTTTCCATCCTCATCCTAATAATTTTACCTCTTACCCACACATCAAAACAACGAAGCTTAATATTCCGACCAATCTCCCAATTTATTTTCTGACTTTTAGTAGCCAACTTACTAATCCTAACATGAATTGGAGGACAACCA

>AK9789-9

GG------------CAGAAGGA----------TACATGCTTACTCTCAGCCATTCCATACATTGGAACAACAATCGTAGAATGAATCTGAGGCGGCTTTTCTGTAGATAATGCCACACTAACACGCTTTTTCGCATTCCATTTTATTCTACCATTTATCATTACTGCTATAGTCCTAGTTCACCTGCTATTTCTCCATGAAACAGGATCTAACAACCCTATAGGCATCAACTCTGACGCAGACAAAATTCCATTCCACCCATATTACACAATCAAAGACCTCTTAGGAGTAATTTTATTAATTTCATTATTCATACTTATAGTCTTATTCTCTCCTGACCTTCTAGGTGACCCCGACAATTACACACCAGCTAACCCACTCAATACACCCCCTCATATCAAGCCAGAATGATACTTCCTATTTGCTTACGCCATTCTACGATCCATCCCTAACAAATTAGGAGGAGTTTTAGCCCTCATTCTTTCCATCCTCATCCTAATAATTTTACCTCTTACCCACACATCAAAACAACGAAGCTTAATATTCCGACCAATCTCCCAATTTATTTTCTGACTTTTAGTAGCCAACTTACTAATCCTAACATGAATTGGAGGACAACCA

>AK9789-8

GG------------CAGAAGGA----------TACATGCTTACTCTCAGCCATTCCATACATTGGAACAACAATCGTAGAATGAATCTGAGGCGGCTTTTCTGTAGATAATGCCACACTAACACGCTTTTTCGCATTCCATTTTATTCTACCATTTATCATTACTGCTATAGTCCTAGTTCACCTGCTATTTCTCCATGAAACAGGATCTAACAACCCTATAGGCATCAACTCTGACGCAGACAAAATTCCATTCCACCCATATTACACAATCAAAGACCTCTTAGGAGTAATTTTATTAATTTCATTATTCATACTTATAGTCTTATTCTCTCCTGACCTTCTAGGTGACCCCGACAATTACACACCAGCTAACCCACTCAATACACCCCCTCATATCAAGCCAGAATGATACTTCCTATTTGCTTACGCCATTCTACGATCCATCCCTAACAAATTAGGAGGAGTTTTAGCCCTCATTCTTTCCATCCTCATCCTAATAATTTTACCTCTTACCCACACATCAAAACAACGAAGCTTAATATTCCGACCAATCTCCCAATTTATTTTCTGACTTTTAGTAGCCAACTTACTAATCCTAACATGAATTGGAGGACAACCA

>AK9789-7

GG------------CAGAAGGA----------TACATGCTTACTCTCAGCCATTCCATACATTGGAACAACAATCGTAGAATGAATCTGAGGCGGCTTTTCTGTAGATAATGCCACACTAACACGCTTTTTCGCATTCCATTTTATTCTACCATTTATCATTACTGCTATAGTCCTAGTTCACCTGCTATTTCTCCATGAAACAGGATCTAACAACCCTATAGGCATCAACTCTGACGCAGACAAAATTCCATTCCACCCATATTACACAATCAAAGACCTCTTAGGAGTAATTTTATTAATTTCATTATTCATACTTATAGTCTTATTCTCTCCTGACCTTCTAGGTGACCCCGACAATTACACACCAGCTAACCCACTCAATACACCCCCTCATATCAAGCCAGAATGATACTTCCTATTTGCTTACGCCATTCTACGATCCATCCCTAACAAATTAGGAGGAGTTTTAGCCCTCATTCTTTCCATCCTCATCCTAATAATTTTACCTCTTACCCACACATCAAAACAACGAAGCTTAATATTCCGACCAATCTCCCAATTTATTTTCTGACTTTTAGTAGCCAACTTACTAATCCTAACATGAATTGGAGGACAACCA

>AK9789-6

GG------------CAGAAGGA----------TACATGCTTACTCTCAGCCATTCCATACATTGGAACAACAATCGTAGAATGAATCTGAGGCGGCTTTTCTGTAGATAATGCCACACTAACACGCTTTTTCGCATTCCATTTTATTCTACCATTTATCATTACTGCTATAGTCCTAGTTCACCTGCTATTTCTCCATGAAACAGGATCTAACAACCCTATAGGCATCAACTCTGACGCAGACAAAATTCCATTCCACCCATATTACACAATCAAAGACCTCTTAGGAGTAATTTTATTAATTTCATTATTCATACTTATAGTCTTATTCTCTCCTGACCTTCTAGGTGACCCCGACAATTACACACCAGCTAACCCACTCAATACACCCCCTCATATCAAGCCAGAATGATACTTCCTATTTGCTTACGCCATTCTACGATCCATCCCTAACAAATTAGGAGGAGTTTTAGCCCTCATTCTTTCCATCCTCATCCTAATAATTTTACCTCTTACCCACACATCAAAACAACGAAGCTTAATATTCCGACCAATCTCCCAATTTATTTTCTGACTTTTAGTAGCCAACTTACTAATCCTAACATGAATTGGAGGACAACCA

>AK9789-5

GG------------CAGAAGGA----------TACATGCTTACTCTCAGCCATTCCATACATTGGAACAACAATCGTAGAATGAATCTGAGGCGGCTTTTCTGTAGATAATGCCACACTAACACGCTTTTTCGCATTCCATTTTATTCTACCATTTATCATTACTGCTATAGTCCTAGTTCACCTGCTATTTCTCCATGAAACAGGATCTAACAACCCTATAGGCATCAACTCTGACGCAGACAAAATTCCATTCCACCCATATTACACAATCAAAGACCTCTTAGGAGTAATTTTATTAATTTCATTATTCATACTTATAGTCTTATTCTCTCCTGACCTTCTAGGTGACCCCGACAATTACACACCAGCTAACCCACTCAATACACCCCCTCATATCAAGCCAGAATGATACTTCCTATTTGCTTACGCCATTCTACGATCCATCCCTAACAAATTAGGAGGAGTTTTAGCCCTCATTCTTTCCATCCTCATCCTAATAATTTTACCTCTTACCCACACATCAAAACAACGAAGCTTAATATTCCGACCAATCTCCCAATTTATTTTCTGACTTTTAGTAGCCAACTTACTAATCCTAACATGAATTGGAGGACAACCA

>AK9789-4

GG------------CAGAAGGA----------TACATGCTTACTCTCAGCCATTCCATACATTGGAACAACAATCGTAGAATGAATCTGAGGCGGCTTTTCTGTAGATAATGCCACACTAACACGCTTTTTCGCATTCCATTTTATTCTACCATTTATCATTACTGCTATAGTCCTAGTTCACCTGCTATTTCTCCATGAAACAGGATCTAACAACCCTATAGGCATCAACTCTGACGCAGACAAAATTCCATTCCACCCATATTACACAATCAAAGACCTCTTAGGAGTAATTTTATTAATTTCATTATTCATACTTATAGTCTTATTCTCTCCTGACCTTCTAGGTGACCCCGACAATTACACACCAGCTAACCCACTCAATACACCCCCTCATATCAAGCCAGAATGATACTTCCTATTTGCTTACGCCATTCTACGATCCATCCCTAACAAATTAGGAGGAGTTTTAGCCCTCATTCTTTCCATCCTCATCCTAATAATTTTACCTCTTACCCACACATCAAAACAACGAAGCTTAATATTCCGACCAATCTCCCAATTTATTTTCTGACTTTTAGTAGCCAACTTACTAATCCTAACATGAATTGGAGGACAACCA

>AK9789-3

GG------------CAGAAGGA----------TACATGCTTACTCTCAGCCATTCCATACATTGGAACAACAATCGTAGAATGAATCTGAGGCGGCTTTTCTGTAGATAATGCCACACTAACACGCTTTTTCGCATTCCATTTTATTCTACCATTTATCATTACTGCTATAGTCCTAGTTCACCTGCTATTTCTCCATGAAACAGGATCTAACAACCCTATAGGCATCAACTCTGACGCAGACAAAATTCCATTCCACCCATATTACACAATCAAAGACCTCTTAGGAGTAATTTTATTAATTTCATTATTCATACTTATAGTCTTATTCTCTCCTGACCTTCTAGGTGACCCCGACAATTACACACCAGCTAACCCACTCAATACACCCCCTCATATCAAGCCAGAATGATACTTCCTATTTGCTTACGCCATTCTACGATCCATCCCTAACAAATTAGGAGGAGTTTTAGCCCTCATTCTTTCCATCCTCATCCTAATAATTTTACCTCTTACCCACACATCAAAACAACGAAGCTTAATATTCCGACCAATCTCCCAATTTATTTTCTGACTTTTAGTAGCCAACTTACTAATCCTAACATGAATTGGAGGACAACCA

>AK9789-2

GG------------CAGAAGGA----------TACATGCTTACTCTCAGCCATTCCATACATTGGAACAACAATCGTAGAATGAATCTGAGGCGGCTTTTCTGTAGATAATGCCACACTAACACGCTTTTTCGCATTCCATTTTATTCTACCATTTATCATTACTGCTATAGTCCTAGTTCACCTGCTATTTCTCCATGAAACAGGATCTAACAACCCTATAGGCATCAACTCTGACGCAGACAAAATTCCATTCCACCCATATTACACAATCAAAGACCTCTTAGGAGTAATTTTATTAATTTCATTATTCATACTTATAGTCTTATTCTCTCCTGACCTTCTAGGTGACCCCGACAATTACACACCAGCTAACCCACTCAATACACCCCCTCATATCAAGCCAGAATGATACTTCCTATTTGCTTACGCCATTCTACGATCCATCCCTAACAAATTAGGAGGAGTTTTAGCCCTCATTCTTTCCATCCTCATCCTAATAATTTTACCTCTTACCCACACATCAAAACAACGAAGCTTAATATTCCGACCAATCTCCCAATTTATTTTCTGACTTTTAGTAGCCAACTTACTAATCCTAACATGAATTGGAGGACAACCA

>AK9789-1

GG------------CAGAAGGA----------TACATGCTTACTCTCAGCCATTCCATACATTGGAACAACAATCGTAGAATGAATCTGAGGCGGCTTTTCTGTAGATAATGCCACACTAACACGCTTTTTCGCATTCCATTTTATTCTACCATTTATCATTACTGCTATAGTCCTAGTTCACCTGCTATTTCTCCATGAAACAGGATCTAACAACCCTATAGGCATCAACTCTGACGCAGACAAAATTCCATTCCACCCATATTACACAATCAAAGACCTCTTAGGAGTAATTTTATTAATTTCATTATTCATACTTATAGTCTTATTCTCTCCTGACCTTCTAGGTGACCCCGACAATTACACACCAGCTAACCCACTCAATACACCCCCTCATATCAAGCCAGAATGATACTTCCTATTTGCTTACGCCATTCTACGATCCATCCCTAACAAATTAGGAGGAGTTTTAGCCCTCATTCTTTCCATCCTCATCCTAATAATTTTACCTCTTACCCACACATCAAAACAACGAAGCTTAATATTCCGACCAATCTCCCAATTTATTTTCTGACTTTTAGTAGCCAACTTACTAATCCTAACATGAATTGGAGGACAACCA
