# Supplementary material for: Morphological and molecular characterization of Tatera indica Hardwicke 1807 (Rodentia: Muridae) from Pothwar, Pakistan
Source: Open Life Sci. 2022 Jun 15;17(1):610–5. doi: 10.1515/biol-2022-0063 (PMC9202534; doi:10.1515/biol-2022-0063)
Supplement: Supplementary Material 2 [file biol-2022-0063-Supplementary-2.docx]

CLUSTAL 2.1 Multiple Sequence Alignments

Sequence type explicitly set to DNA

Sequence format is Pearson

Sequence 1: AK9789-13 621 bp

Sequence 2: AK9789-12 621 bp

Sequence 3: AK9789-11 621 bp

Sequence 4: AK9789-10 621 bp

Sequence 5: AK9789-9 621 bp

Sequence 6: AK9789-8 621 bp

Sequence 7: AK9789-7 621 bp

Sequence 8: AK9789-6 621 bp

Sequence 9: AK9789-5 621 bp

Sequence 10: AK9789-4 621 bp

Sequence 11: AK9789-3 621 bp

Sequence 12: AK9789-2 621 bp

Sequence 13: AK9789-1 621 bp

Start of Pairwise alignments

Aligning...

Sequences (1:2) Aligned. Score: 99

Sequences (1:3) Aligned. Score: 99

Sequences (1:4) Aligned. Score: 99

Sequences (1:5) Aligned. Score: 99

Sequences (1:6) Aligned. Score: 99

Sequences (1:7) Aligned. Score: 99

Sequences (1:8) Aligned. Score: 99

Sequences (1:9) Aligned. Score: 99

Sequences (1:10) Aligned. Score: 99

Sequences (1:11) Aligned. Score: 99

Sequences (1:12) Aligned. Score: 99

Sequences (1:13) Aligned. Score: 99

Sequences (2:3) Aligned. Score: 99

Sequences (2:4) Aligned. Score: 99

Sequences (2:5) Aligned. Score: 99

Sequences (2:6) Aligned. Score: 99

Sequences (2:7) Aligned. Score: 99

Sequences (2:8) Aligned. Score: 99

Sequences (2:9) Aligned. Score: 99

Sequences (2:10) Aligned. Score: 99

Sequences (2:11) Aligned. Score: 99

Sequences (2:12) Aligned. Score: 99

Sequences (2:13) Aligned. Score: 99

Sequences (3:4) Aligned. Score: 100

Sequences (3:5) Aligned. Score: 100

Sequences (3:6) Aligned. Score: 100

Sequences (3:7) Aligned. Score: 100

Sequences (3:8) Aligned. Score: 100

Sequences (3:9) Aligned. Score: 100

Sequences (3:10) Aligned. Score: 100

Sequences (3:11) Aligned. Score: 100

Sequences (3:12) Aligned. Score: 100

Sequences (3:13) Aligned. Score: 100

Sequences (4:5) Aligned. Score: 100

Sequences (4:6) Aligned. Score: 100

Sequences (4:7) Aligned. Score: 100

Sequences (4:8) Aligned. Score: 100

Sequences (4:9) Aligned. Score: 100

Sequences (4:10) Aligned. Score: 100

Sequences (4:11) Aligned. Score: 100

Sequences (4:12) Aligned. Score: 100

Sequences (4:13) Aligned. Score: 100

Sequences (5:6) Aligned. Score: 100

Sequences (5:7) Aligned. Score: 100

Sequences (5:8) Aligned. Score: 100

Sequences (5:9) Aligned. Score: 100

Sequences (5:10) Aligned. Score: 100

Sequences (5:11) Aligned. Score: 100

Sequences (5:12) Aligned. Score: 100

Sequences (5:13) Aligned. Score: 100

Sequences (6:7) Aligned. Score: 100

Sequences (6:8) Aligned. Score: 100

Sequences (6:9) Aligned. Score: 100

Sequences (6:10) Aligned. Score: 100

Sequences (6:11) Aligned. Score: 100

Sequences (6:12) Aligned. Score: 100

Sequences (6:13) Aligned. Score: 100

Sequences (7:8) Aligned. Score: 100

Sequences (7:9) Aligned. Score: 100

Sequences (7:10) Aligned. Score: 100

Sequences (7:11) Aligned. Score: 100

Sequences (7:12) Aligned. Score: 100

Sequences (7:13) Aligned. Score: 100

Sequences (8:9) Aligned. Score: 100

Sequences (8:10) Aligned. Score: 100

Sequences (8:11) Aligned. Score: 100

Sequences (8:12) Aligned. Score: 100

Sequences (8:13) Aligned. Score: 100

Sequences (9:10) Aligned. Score: 100

Sequences (9:11) Aligned. Score: 100

Sequences (9:12) Aligned. Score: 100

Sequences (9:13) Aligned. Score: 100

Sequences (10:11) Aligned. Score: 100

Sequences (10:12) Aligned. Score: 100

Sequences (10:13) Aligned. Score: 100

Sequences (11:12) Aligned. Score: 100

Sequences (11:13) Aligned. Score: 100

Sequences (12:13) Aligned. Score: 100

Guide tree file created: [[clustalw.dnd]](https://www.genome.jp/tools-bin/pushfile?220408025912WcyG2+clustalw.dnd)

There are 12 groups

Start of Multiple Alignment

Aligning...

Group 1: Sequences: 2 Score:11381

Group 2: Sequences: 3 Score:11381

Group 3: Sequences: 4 Score:11381

Group 4: Sequences: 5 Score:11381

Group 5: Sequences: 6 Score:11381

Group 6: Sequences: 7 Score:11381

Group 7: Sequences: 8 Score:11381

Group 8: Sequences: 9 Score:11381

Group 9: Sequences: 10 Score:11381

Group 10: Sequences: 11 Score:11381

Group 11: Sequences: 12 Score:11371

Group 12: Sequences: 13 Score:11361

Alignment Score 333618

CLUSTAL-Alignment file created [[clustalw.aln]](https://www.genome.jp/tools-bin/pushfile?220408025912WcyG2+clustalw.aln)

[clustalw.aln](https://www.genome.jp/tools-bin/pushfile?220408025912WcyG2+clustalw.aln)

CLUSTAL 2.1 multiple sequence alignment

AK9789-2 GG------------CAGAAGGA----------TACATGCTTACTCTCAGCCATTCCATAC

AK9789-1 GG------------CAGAAGGA----------TACATGCTTACTCTCAGCCATTCCATAC

AK9789-3 GG------------CAGAAGGA----------TACATGCTTACTCTCAGCCATTCCATAC

AK9789-4 GG------------CAGAAGGA----------TACATGCTTACTCTCAGCCATTCCATAC

AK9789-5 GG------------CAGAAGGA----------TACATGCTTACTCTCAGCCATTCCATAC

AK9789-6 GG------------CAGAAGGA----------TACATGCTTACTCTCAGCCATTCCATAC

AK9789-7 GG------------CAGAAGGA----------TACATGCTTACTCTCAGCCATTCCATAC

AK9789-8 GG------------CAGAAGGA----------TACATGCTTACTCTCAGCCATTCCATAC

AK9789-9 GG------------CAGAAGGA----------TACATGCTTACTCTCAGCCATTCCATAC

AK9789-10 GG------------CAGAAGGA----------TACATGCTTACTCTCAGCCATTCCATAC

AK9789-11 GG------------CAGAAGGA----------TACATGCTTACTCTCAGCCATTCCATAC

AK9789-13 GG------------CAGAAGGA----------TACATGCTTACTCTCAGCCATTCCATAC

AK9789-12 GG------------CAGAAGGA----------TACATGCTTACTCTCAGCCATTCCATAC

** ******** ****************************

AK9789-2 ATTGGAACAACAATCGTAGAATGAATCTGAGGCGGCTTTTCTGTAGATAATGCCACACTA

AK9789-1 ATTGGAACAACAATCGTAGAATGAATCTGAGGCGGCTTTTCTGTAGATAATGCCACACTA

AK9789-3 ATTGGAACAACAATCGTAGAATGAATCTGAGGCGGCTTTTCTGTAGATAATGCCACACTA

AK9789-4 ATTGGAACAACAATCGTAGAATGAATCTGAGGCGGCTTTTCTGTAGATAATGCCACACTA

AK9789-5 ATTGGAACAACAATCGTAGAATGAATCTGAGGCGGCTTTTCTGTAGATAATGCCACACTA

AK9789-6 ATTGGAACAACAATCGTAGAATGAATCTGAGGCGGCTTTTCTGTAGATAATGCCACACTA

AK9789-7 ATTGGAACAACAATCGTAGAATGAATCTGAGGCGGCTTTTCTGTAGATAATGCCACACTA

AK9789-8 ATTGGAACAACAATCGTAGAATGAATCTGAGGCGGCTTTTCTGTAGATAATGCCACACTA

AK9789-9 ATTGGAACAACAATCGTAGAATGAATCTGAGGCGGCTTTTCTGTAGATAATGCCACACTA

AK9789-10 ATTGGAACAACAATCGTAGAATGAATCTGAGGCGGCTTTTCTGTAGATAATGCCACACTA

AK9789-11 ATTGGAACAACAATCGTAGAATGAATCTGAGGCGGCTTTTCTGTAGATAATGCCACACTA

AK9789-13 ATTGGAACAACAATCGTAGAATGAATCTGAGGCGGCTTTTCTGTAGATAATGCCACACTA

AK9789-12 ATTGGAACAACAATCGTAGAATGAATCTGAGGCGGCTTTTCTGTAGATAATGCCACACTA

************************************************************

AK9789-2 ACACGCTTTTTCGCATTCCATTTTATTCTACCATTTATCATTACTGCTATAGTCCTAGTT

AK9789-1 ACACGCTTTTTCGCATTCCATTTTATTCTACCATTTATCATTACTGCTATAGTCCTAGTT

AK9789-3 ACACGCTTTTTCGCATTCCATTTTATTCTACCATTTATCATTACTGCTATAGTCCTAGTT

AK9789-4 ACACGCTTTTTCGCATTCCATTTTATTCTACCATTTATCATTACTGCTATAGTCCTAGTT

AK9789-5 ACACGCTTTTTCGCATTCCATTTTATTCTACCATTTATCATTACTGCTATAGTCCTAGTT

AK9789-6 ACACGCTTTTTCGCATTCCATTTTATTCTACCATTTATCATTACTGCTATAGTCCTAGTT

AK9789-7 ACACGCTTTTTCGCATTCCATTTTATTCTACCATTTATCATTACTGCTATAGTCCTAGTT

AK9789-8 ACACGCTTTTTCGCATTCCATTTTATTCTACCATTTATCATTACTGCTATAGTCCTAGTT

AK9789-9 ACACGCTTTTTCGCATTCCATTTTATTCTACCATTTATCATTACTGCTATAGTCCTAGTT

AK9789-10 ACACGCTTTTTCGCATTCCATTTTATTCTACCATTTATCATTACTGCTATAGTCCTAGTT

AK9789-11 ACACGCTTTTTCGCATTCCATTTTATTCTACCATTTATCATTACTGCTATAGTCCTAGTT

AK9789-13 ACACGCTTTTTCGCATTCCATTTTATTCTACCATTTATCATTACTGCTATAGTCCTAGTT

AK9789-12 ACACGCTTTTTCGCATTCCATTTTATTTTACCATTTATCATCACTGCTATAGTCCTAGTT

*************************** ************* ******************

AK9789-2 CACCTGCTATTTCTCCATGAAACAGGATCTAACAACCCTATAGGCATCAACTCTGACGCA

AK9789-1 CACCTGCTATTTCTCCATGAAACAGGATCTAACAACCCTATAGGCATCAACTCTGACGCA

AK9789-3 CACCTGCTATTTCTCCATGAAACAGGATCTAACAACCCTATAGGCATCAACTCTGACGCA

AK9789-4 CACCTGCTATTTCTCCATGAAACAGGATCTAACAACCCTATAGGCATCAACTCTGACGCA

AK9789-5 CACCTGCTATTTCTCCATGAAACAGGATCTAACAACCCTATAGGCATCAACTCTGACGCA

AK9789-6 CACCTGCTATTTCTCCATGAAACAGGATCTAACAACCCTATAGGCATCAACTCTGACGCA

AK9789-7 CACCTGCTATTTCTCCATGAAACAGGATCTAACAACCCTATAGGCATCAACTCTGACGCA

AK9789-8 CACCTGCTATTTCTCCATGAAACAGGATCTAACAACCCTATAGGCATCAACTCTGACGCA

AK9789-9 CACCTGCTATTTCTCCATGAAACAGGATCTAACAACCCTATAGGCATCAACTCTGACGCA

AK9789-10 CACCTGCTATTTCTCCATGAAACAGGATCTAACAACCCTATAGGCATCAACTCTGACGCA

AK9789-11 CACCTGCTATTTCTCCATGAAACAGGATCTAACAACCCTATAGGCATCAACTCTGACGCA

AK9789-13 CACCTGCTATTTCTCCATGAAACAGGATCTAACAACCCTATAGGCATCAACTCTGACGCA

AK9789-12 CACCTGCTATTTCTCCATGAAACAGGATCTAACAACCCTATAGGCATCAACTCTGACGCA

************************************************************

AK9789-2 GACAAAATTCCATTCCACCCATATTACACAATCAAAGACCTCTTAGGAGTAATTTTATTA

AK9789-1 GACAAAATTCCATTCCACCCATATTACACAATCAAAGACCTCTTAGGAGTAATTTTATTA

AK9789-3 GACAAAATTCCATTCCACCCATATTACACAATCAAAGACCTCTTAGGAGTAATTTTATTA

AK9789-4 GACAAAATTCCATTCCACCCATATTACACAATCAAAGACCTCTTAGGAGTAATTTTATTA

AK9789-5 GACAAAATTCCATTCCACCCATATTACACAATCAAAGACCTCTTAGGAGTAATTTTATTA

AK9789-6 GACAAAATTCCATTCCACCCATATTACACAATCAAAGACCTCTTAGGAGTAATTTTATTA

AK9789-7 GACAAAATTCCATTCCACCCATATTACACAATCAAAGACCTCTTAGGAGTAATTTTATTA

AK9789-8 GACAAAATTCCATTCCACCCATATTACACAATCAAAGACCTCTTAGGAGTAATTTTATTA

AK9789-9 GACAAAATTCCATTCCACCCATATTACACAATCAAAGACCTCTTAGGAGTAATTTTATTA

AK9789-10 GACAAAATTCCATTCCACCCATATTACACAATCAAAGACCTCTTAGGAGTAATTTTATTA

AK9789-11 GACAAAATTCCATTCCACCCATATTACACAATCAAAGACCTCTTAGGAGTAATTTTATTA

AK9789-13 GACAAAATTCCATTCCACCCATATTACACAATCAAAGACCTCTTAGGAGTAATTTTATTA

AK9789-12 GACAAAATTCCATTCCACCCATATTACACAATCAAAGACCTCTTAGGAGTAATTTTATTA

************************************************************

AK9789-2 ATTTCATTATTCATACTTATAGTCTTATTCTCTCCTGACCTTCTAGGTGACCCCGACAAT

AK9789-1 ATTTCATTATTCATACTTATAGTCTTATTCTCTCCTGACCTTCTAGGTGACCCCGACAAT

AK9789-3 ATTTCATTATTCATACTTATAGTCTTATTCTCTCCTGACCTTCTAGGTGACCCCGACAAT

AK9789-4 ATTTCATTATTCATACTTATAGTCTTATTCTCTCCTGACCTTCTAGGTGACCCCGACAAT

AK9789-5 ATTTCATTATTCATACTTATAGTCTTATTCTCTCCTGACCTTCTAGGTGACCCCGACAAT

AK9789-6 ATTTCATTATTCATACTTATAGTCTTATTCTCTCCTGACCTTCTAGGTGACCCCGACAAT

AK9789-7 ATTTCATTATTCATACTTATAGTCTTATTCTCTCCTGACCTTCTAGGTGACCCCGACAAT

AK9789-8 ATTTCATTATTCATACTTATAGTCTTATTCTCTCCTGACCTTCTAGGTGACCCCGACAAT

AK9789-9 ATTTCATTATTCATACTTATAGTCTTATTCTCTCCTGACCTTCTAGGTGACCCCGACAAT

AK9789-10 ATTTCATTATTCATACTTATAGTCTTATTCTCTCCTGACCTTCTAGGTGACCCCGACAAT

AK9789-11 ATTTCATTATTCATACTTATAGTCTTATTCTCTCCTGACCTTCTAGGTGACCCCGACAAT

AK9789-13 ATTTCATTATTCATACTTATAGTCCTATTCTCTCCTGACCTTCTAGGTGACCCCGACAAT

AK9789-12 ATTTCATTATTCATACTTATAGTCTTATTCTCTCCTGACCTTCTAGGTGACCCCGACAAT

************************ ***********************************

AK9789-2 TACACACCAGCTAACCCACTCAATACACCCCCTCATATCAAGCCAGAATGATACTTCCTA

AK9789-1 TACACACCAGCTAACCCACTCAATACACCCCCTCATATCAAGCCAGAATGATACTTCCTA

AK9789-3 TACACACCAGCTAACCCACTCAATACACCCCCTCATATCAAGCCAGAATGATACTTCCTA

AK9789-4 TACACACCAGCTAACCCACTCAATACACCCCCTCATATCAAGCCAGAATGATACTTCCTA

AK9789-5 TACACACCAGCTAACCCACTCAATACACCCCCTCATATCAAGCCAGAATGATACTTCCTA

AK9789-6 TACACACCAGCTAACCCACTCAATACACCCCCTCATATCAAGCCAGAATGATACTTCCTA

AK9789-7 TACACACCAGCTAACCCACTCAATACACCCCCTCATATCAAGCCAGAATGATACTTCCTA

AK9789-8 TACACACCAGCTAACCCACTCAATACACCCCCTCATATCAAGCCAGAATGATACTTCCTA

AK9789-9 TACACACCAGCTAACCCACTCAATACACCCCCTCATATCAAGCCAGAATGATACTTCCTA

AK9789-10 TACACACCAGCTAACCCACTCAATACACCCCCTCATATCAAGCCAGAATGATACTTCCTA

AK9789-11 TACACACCAGCTAACCCACTCAATACACCCCCTCATATCAAGCCAGAATGATACTTCCTA

AK9789-13 TACACACCAGCTAACCCACTCAATACACCCCCTCATATCAAGCCAGAATGATACTTCCTA

AK9789-12 TACACACCAGCTAACCCACTCAATACACCCCCTCATATCAAGCCAGAATGATACTTCCTA

************************************************************

AK9789-2 TTTGCTTACGCCATTCTACGATCCATCCCTAACAAATTAGGAGGAGTTTTAGCCCTCATT

AK9789-1 TTTGCTTACGCCATTCTACGATCCATCCCTAACAAATTAGGAGGAGTTTTAGCCCTCATT

AK9789-3 TTTGCTTACGCCATTCTACGATCCATCCCTAACAAATTAGGAGGAGTTTTAGCCCTCATT

AK9789-4 TTTGCTTACGCCATTCTACGATCCATCCCTAACAAATTAGGAGGAGTTTTAGCCCTCATT

AK9789-5 TTTGCTTACGCCATTCTACGATCCATCCCTAACAAATTAGGAGGAGTTTTAGCCCTCATT

AK9789-6 TTTGCTTACGCCATTCTACGATCCATCCCTAACAAATTAGGAGGAGTTTTAGCCCTCATT

AK9789-7 TTTGCTTACGCCATTCTACGATCCATCCCTAACAAATTAGGAGGAGTTTTAGCCCTCATT

AK9789-8 TTTGCTTACGCCATTCTACGATCCATCCCTAACAAATTAGGAGGAGTTTTAGCCCTCATT

AK9789-9 TTTGCTTACGCCATTCTACGATCCATCCCTAACAAATTAGGAGGAGTTTTAGCCCTCATT

AK9789-10 TTTGCTTACGCCATTCTACGATCCATCCCTAACAAATTAGGAGGAGTTTTAGCCCTCATT

AK9789-11 TTTGCTTACGCCATTCTACGATCCATCCCTAACAAATTAGGAGGAGTTTTAGCCCTCATT

AK9789-13 TTTGCTTACGCCATTCTACGATCCATCCCTAACAAATTAGGAGGAGTTTTAGCCCTCATT

AK9789-12 TTTGCTTACGCCATTCTACGATCCATCCCTAACAAATTAGGAGGAGTTTTAGCCCTCATT

************************************************************

AK9789-2 CTTTCCATCCTCATCCTAATAATTTTACCTCTTACCCACACATCAAAACAACGAAGCTTA

AK9789-1 CTTTCCATCCTCATCCTAATAATTTTACCTCTTACCCACACATCAAAACAACGAAGCTTA

AK9789-3 CTTTCCATCCTCATCCTAATAATTTTACCTCTTACCCACACATCAAAACAACGAAGCTTA

AK9789-4 CTTTCCATCCTCATCCTAATAATTTTACCTCTTACCCACACATCAAAACAACGAAGCTTA

AK9789-5 CTTTCCATCCTCATCCTAATAATTTTACCTCTTACCCACACATCAAAACAACGAAGCTTA

AK9789-6 CTTTCCATCCTCATCCTAATAATTTTACCTCTTACCCACACATCAAAACAACGAAGCTTA

AK9789-7 CTTTCCATCCTCATCCTAATAATTTTACCTCTTACCCACACATCAAAACAACGAAGCTTA

AK9789-8 CTTTCCATCCTCATCCTAATAATTTTACCTCTTACCCACACATCAAAACAACGAAGCTTA

AK9789-9 CTTTCCATCCTCATCCTAATAATTTTACCTCTTACCCACACATCAAAACAACGAAGCTTA

AK9789-10 CTTTCCATCCTCATCCTAATAATTTTACCTCTTACCCACACATCAAAACAACGAAGCTTA

AK9789-11 CTTTCCATCCTCATCCTAATAATTTTACCTCTTACCCACACATCAAAACAACGAAGCTTA

AK9789-13 CTTTCCATCCTCATCCTAATAATTTTACCTCTTACCCACACATCAAAACAACGAAGCTTA

AK9789-12 CTTTCCATCCTCATCCTAATAATTTTACCTCTTACCCACACATCAAAACAACGAAGCTTA

************************************************************

AK9789-2 ATATTCCGACCAATCTCCCAATTTATTTTCTGACTTTTAGTAGCCAACTTACTAATCCTA

AK9789-1 ATATTCCGACCAATCTCCCAATTTATTTTCTGACTTTTAGTAGCCAACTTACTAATCCTA

AK9789-3 ATATTCCGACCAATCTCCCAATTTATTTTCTGACTTTTAGTAGCCAACTTACTAATCCTA

AK9789-4 ATATTCCGACCAATCTCCCAATTTATTTTCTGACTTTTAGTAGCCAACTTACTAATCCTA

AK9789-5 ATATTCCGACCAATCTCCCAATTTATTTTCTGACTTTTAGTAGCCAACTTACTAATCCTA

AK9789-6 ATATTCCGACCAATCTCCCAATTTATTTTCTGACTTTTAGTAGCCAACTTACTAATCCTA

AK9789-7 ATATTCCGACCAATCTCCCAATTTATTTTCTGACTTTTAGTAGCCAACTTACTAATCCTA

AK9789-8 ATATTCCGACCAATCTCCCAATTTATTTTCTGACTTTTAGTAGCCAACTTACTAATCCTA

AK9789-9 ATATTCCGACCAATCTCCCAATTTATTTTCTGACTTTTAGTAGCCAACTTACTAATCCTA

AK9789-10 ATATTCCGACCAATCTCCCAATTTATTTTCTGACTTTTAGTAGCCAACTTACTAATCCTA

AK9789-11 ATATTCCGACCAATCTCCCAATTTATTTTCTGACTTTTAGTAGCCAACTTACTAATCCTA

AK9789-13 ATATTCCGACCAATCTCCCAATTTATTTTCTGACTTTTAGTAGCCAACTTACTAATCCTA

AK9789-12 ATATTCCGACCAATCTCCCAATTTATTTTCTGACTTTTAGTAGCCAACTTACTAATCCTA

************************************************************

AK9789-2 ACATGAATTGGAGGACAACCA

AK9789-1 ACATGAATTGGAGGACAACCA

AK9789-3 ACATGAATTGGAGGACAACCA

AK9789-4 ACATGAATTGGAGGACAACCA

AK9789-5 ACATGAATTGGAGGACAACCA

AK9789-6 ACATGAATTGGAGGACAACCA

AK9789-7 ACATGAATTGGAGGACAACCA

AK9789-8 ACATGAATTGGAGGACAACCA

AK9789-9 ACATGAATTGGAGGACAACCA

AK9789-10 ACATGAATTGGAGGACAACCA

AK9789-11 ACATGAATTGGAGGACAACCA

AK9789-13 ACATGAATTGGAGGACAACCA

AK9789-12 ACATGAATTGGAGGACAACCA

*********************
